# Everybody's Page

**Published:** 1889-03-30

**Authors:** 


### Everybody's Page.

The hours during which it is safe for invalids to venture out, constitute what is called on the Riviera the "medical" day.

In Russia, everyone found inebriated in the public streets is imprisoned, and when he has recovered from his intoxication is set to sweep the streets for a day. It is thus not rare to see in the streets of St. Petersburg well-dressed men acting thus as scavengers.

Surgeon-Major Yvert, who lately returned to France from Tonquin, reports the successful use of inoculations of bi-chloride of mercury in cholera. The average mortality from cholera in that part of Asia is 66 per cent. In Dr. Yvert's practice the cases where he employed his new treatment showed a mortality of only 20 per cent.

A French engineer, M. Desmarets, recommends the institution of what he calls "ambulance kiosques." He would like to see 300 kiosques set up in the most frequented streets of Paris, supplied with simple medicines, dressings, and everything necessary to render first aid to the injured, as well as the necessary means of conveying the patient to the hospital.

Tea-drinking in excess, especially when the tea has been allowed to "draw" for a considerable time, produces a sensation of dryness in the throat, the result of dyspepsia. Dr. Walker Downie found that the substitution of cocoa or milk for the tea, accompanied by the administration of an iron tonic, not only cured the ailment, but caused the patients to take a dislike to the stewed tea.

No one familiar with doctors would insinuate that they do not generally display the homely but useful quality of common sense. But it is undoubtedly true that many members of the so called "learned professions" are terribly crushed and weighed down with the burden of their professional distinctions. It is almost impossible under any circumstances to make a clergyman, or a lawyer, or a doctor forget that he is a "professional man." There is a good deal of stupidity in this, and, not seldom, danger. The doctor, for example, when applied to professionally, thinks he must always do that which professional feeling and interest would dictate; whereas it would often be better if he would follow the higher instincts of a generous manhood, rather than the lower ones of mere professional business. This little preface is occasioned by the satisfaction we feel in finding that Mr. Priestley Smith, ophthalmic surgeon to the Queen's Hospital, Birmingham, has not given way to the modern "spectacles craze," which is so rampant among the medical guardians of school children in London and elsewhere. It may be presumed that the "eye troubles" of this class of children have been pressing themselves upon the school managers at Birmingham lately, and that professional attention has therefore been given to the subject. Mr. Priestley Smith has not indiscriminately prescribed the universal panacea, which is often so useless and always so absurd-looking in children, viz., spectacles. On the contrary, he goes to the root of the matter, and resolves to do what he can to *prevent* eye mischief, knowing full well that prevented diseases never need costly cures. He has formulated seven rules for children, which are well worthy of the designation "golden," and which every schoolmaster should hang up in his school in the biggest type he can find. Here are the rules, brief, plain, pointed: "Sit upright and sit square; keep your eyes at least twelve inches from your work; write on a slope and not on a flat table; read with your book well up; do not read very small print; do not work in a bad light; if you cannot see your work properly, tell your teacher." To all of which every rational doctor will, in the words of the Prayer-book, give his "unfeigned assent and consent," and his most emphatic commendation.

The deaths from typhoid fever in the French Army during 1885 were in the proportion of 53 in every 1,000 cases.

Dr. Salemi, of Nice, recommends strong carbolic acid for the cure of corns. After bathing the feet, melt the carbolic crystals and apply a thick layer to the corn, taking care not to touch the sound skin round it. After a few minutes, apply a piece of blotting-paper or cotton-wool to the acid to absorb any excess. Repeated every three or four days, this is said to be a certain cure.

The pilgrimage to Mecca, which all good Mahommedans make at least once in their lives, is a fruitful source of disease and death. Last year about 200,000 pilgrims visited the shrines at Mecca and Medina. The road between these two towns is bad, the heat is overpowering, and water is scarce; yet such is the energy of their fanaticism that aged men, invalids, and delicate women attempt the journey. All the pilgrims, indeed, are not impelled by religious motives. There are among them vagabonds and robbers, who steal from their fellows, and do not even shrink from murdering them if need be. Some years ago the Turkish Government spent large sums in attending to the wells and aqueducts of the district, but the Arabs have destroyed these, to prevent the pilgrims getting supplies of water without paying for it. The vessels in which the pilgrims came to the shores of Arabia from every quarter of Islam, used also to be absolutely plague-ships, where small-pox and cholera ran riot; but of late years, especially when ships under English control are used, there has been a great improvement in this matter. Last year, among 1,489 pilgrims who went from Singapore there occurred only two cases of small-pox, neither of which was fatal, and none of cholera. Thirteen deaths occurred, chiefly from consumption or old age.

We have already expressed the opinion more than once, that no cautious person with a high sense of public duty could continue to subscribe to St. John's Hospital for Diseases of the Skin, until those responsible for its management had consented to allow its administration to be impartially and thoroughly investigated by a competent authority. This view was endorsed in the Court of Queen's Bench by the Lord Chief Justice (Lord Coleridge) on Monday. Lord Coleridge asked, "Could it be much wondered at that people outside, who did not know anything about the matter—and all that they did know was irregularity on the one hand, and refusal of investigation on the other—should use somewhat strong language? So far as he, Lord Coleridge, had any unfavourable impression towards the plaintiff in the action of Mercier *v.* Labouchere, it was from plaintiff's own statements, and it was not probable that that impression could be made less unfavourable by cross-examination. Here was a gentleman whose first duty was to receive the cheques and pay out the money. He had got himself into a mode of conduct of carrying on affairs, and continued the practices for a long time, until at last they were found out. They were practices he must have felt it not very easy to disclose, and he was obliged to hide it from observation by falsehood, but it was much to his credit that he, as a gentleman, admitted the falsehood. An inquiry had been obstinately refused, and the defendant, having an honest wish that this institution should be conducted properly, had used these expressions ("dishonest practices"). He did not like to give ignominious damages, but he thought the defendant had been very much led by the plaintiff to use these expressions, and that the plaintiff and those who stood by him in the management of the hospital were very much to blame. His judgment on the matter would be that "the defendant pay forty shillings, and no more." In these circumstances we must again emphatically declare, that unless an independent inquiry into the whole management of this hospital is at once instituted, we hope, in the public interests, that it may be speedily closed for want of pecuniary support.

